# Coverage, compliance, acceptability and feasibility of a program to prevent pre-eclampsia and eclampsia through calcium supplementation for pregnant women: an operations research study in one district of Nepal

**DOI:** 10.1186/s12884-016-1033-6

**Published:** 2016-08-24

**Authors:** Kusum Thapa, Harshad Sanghvi, Barbara Rawlins, Yagya B. Karki, Kiran Regmi, Shilu Aryal, Yeshoda Aryal, Peter Murakami, Jona Bhattarai, Stephanie Suhowatsky

**Affiliations:** 1 Jhpiego/Nepal, Oasis Building, Patan Dhoka, Lalitpur, Kathmandu Nepal; 2 Jhpiego, 1615 Thames Street, Baltimore, MD 23121 USA; 3 Maternal and Child Survival Program/Jhpiego, 1776 Massachusetts Avenue, Washington, DC 20036 USA; 4 The Population, Health and Development (PHD) Group, Sanepa, Lalitpur, Kathmandu Nepal; 5 Ministry of Health and Population, Government of Nepal, Teku, Kathmandu Nepal; 6 Department of Biostatistics, Johns Hopkins University Bloomberg School of Public Health, 615 N. Wolfe St, Baltimore, MD 21205 USA

**Keywords:** Calcium, Pre-eclampsia, Eclampsia prevention, Maternal health, Coverage, Compliance, Acceptability, Feasibility, Antenatal care

## Abstract

**Background:**

Calcium supplementation during pregnancy has been shown to reduce the incidence of pre-eclampsia/eclampsia among women with low calcium intake. Universal free calcium supplementation through government antenatal care (ANC) services was piloted in the Dailekh district of Nepal. Coverage, compliance, acceptability and feasibility of the intervention were evaluated.

**Methods:**

Antenatal care providers were trained to distribute and counsel pregnant women about calcium use, and female community health volunteers (FCHVs) were trained to reinforce calcium-related messages. A post-intervention cluster household survey was conducted among women who had given birth in the last six months. Secondary data analysis was performed using monitoring data from health facilities and FCHVs.

**Results:**

One Thousand Two hundred-forty postpartum women were interviewed. Most (94.6 %) had attended at least one ANC visit; the median gestational age at first ANC visit was 4 months. All who attended ANC were counseled about calcium and received calcium tablets to take daily until delivery.79.5 % of the women reported consuming the entire quantity of calcium they received. The full course of calcium (300 tablets for 150 days) was provided to 82.3 % of the women. Consumption of the full course of calcium was reported by 67.3 % of all calcium recipients. Significant predictors of completing a full course were gestational age at first ANC visit and number of ANC visits during their most recent pregnancy (*p* < 0.01). Nearly all (99.2 %) reported taking the calcium as instructed with respect to dose, timing and frequency. Among women who received both calcium and iron (*n* = 1,157), 98.0 % reported taking them at different times of the day, as instructed. Over 97 % reported willingness to recommend calcium to others, and said they would like to use it during a subsequent pregnancy. There were no stock-outs of calcium.

**Conclusions:**

Calcium distribution through ANC was feasible and effective, achieving 94.6 % calcium coverage of pregnant women in the district. Most women (over 80 %) attended ANC early enough in pregnancy to receive the full course of calcium supplements and benefit from the intervention. High coverage, compliance, acceptability among pregnant women and feasibility were reported, suggesting that this intervention can be scaled up in other areas of Nepal.

## Background

Pre-eclampsia/eclampsia(PE/E) and other hypertensive disorders during pregnancy cause 14 % of maternal deaths globally [1]. The incidence of pre-eclampsia is estimated at 3.2 % of live births, or a total number of more than four million cases each year, of which more than 72,000 were fatal [2]. Hypertensive disorders can also result in pre-term birth, a leading cause of neonatal mortality [3]. There are no reliable predictive tests for the development of pre-eclampsia or the progression to eclampsia [4], so greater attention is being given to interventions to prevent PE/E. Recent clinical trials indicate that calcium supplementation with doses of at least 1 g of elemental calcium per day during pregnancy more than halved the risk of pre-eclampsia [5]. The World Health Organization recommends calcium supplementation for all women in areas where dietary calcium intake is low and for those at high risk of developing pre-eclampsia [6].

In Nepal, as the maternal mortality ratio has decreased, eclampsia has become the leading cause of maternal deaths [7], replacing postpartum hemorrhage. To try to address the problem, the Nepal Ministry of Health and Population (MOHP) has evidence-based clinical standards in place for PE/E screening during antenatal care (ANC) (i.e., routine urine testing for protein; blood pressure measurement) and PE/E management at all health facilities. ANC attendance in Nepal increased from 49.1 % in 2001 [8] to 84.8 % in 2011 [9] for at least one visit. In 2011, almost half of ANC clients (49.7 %) came for their first visit before 4 months, and 50.1 % of women who had recently delivered attended the recommended four or more ANC visits [9], indicating increasing opportunities to screen and treat women for PE/E during pregnancy. The MOHP and national stakeholders recognize there is a lack of information on calcium deficiency among women in Nepal and a greater need for calcium during pregnancy and while breastfeeding to prevent PE/E and preterm births and to address maternal under nutrition [10].

As the MOHP explored the possibility of introducing calcium supplementation during pregnancy, they found a lack of programmatic evidence about the best approaches for reaching pregnant women and the acceptability of supplements among pregnant women and their families. Acceptance of and compliance with daily calcium consumption among pregnant women outside of clinical trials were unknown. The MOHP, with assistance from the Maternal and Child Health Integrated Program (MCHIP) and Jhpiego, therefore designed an operations research study in one district of Nepal to evaluate whether integration of free calcium supplementation with existing government ANC services was feasible and effective. Jhpiego, an international non-profit health organization affiliated with the Johns Hopkins University, is working in Nepal to support Ministry of Health (MoH) especially in maternal health. The primary objectives of the study were to assess the coverage, compliance, acceptability and feasibility of the antenatal calcium supplementation intervention. The study was not designed to measure the incidence of PE/E or clinical efficacy of calcium supplementation, given the clinical evidence already documented [3]. Rather, it was intended to generate information to inform policy decisions by the MOHP regarding scale-up of the intervention to other districts in Nepal.

## Methods

### Study setting

Dailekh district, a large hill district in the Bheri zone of the Mid-western Region of Nepal, was selected for the study by the Family Health Division of the MOHP in consultation with a technical advisory group. The population is about 261,770, with approximately 48,919 households, 63,073 women of reproductive age, and 7407 expected pregnancies per year [11, 12]. Dailekh has 55 village development committees (VDCs) and one municipality. There is a mix of castes/ethnicities, including highly disadvantaged *Dalit*, disadvantaged/Janajati and upper caste groups. There are government health facilities and 810 female community health volunteers (FCHVs) in Dailekh [12].

### The intervention

ANC was selected as the mechanism for counseling and distribution of calcium to pregnant women in light of the relatively high ANC attendance in Nepal as well as logistical considerations. While Nepal has a strong network of more than 48,000 FCHVs [12] who deliver maternal health information and products (e.g., iron folate supplements, misoprostol) to women at home, calcium is a bulky commodity that would be logistically problematic for FCHVs to carry, given the quantity required for antenatal supplementation.

The antenatal calcium supplementation intervention was defined as follows: (1) education and counseling about PE/E and calcium for all pregnant women who attend ANC (primarily during the first ANC visit), (2) provision of enough calcium carbonate tablets to last for 5 months or until the end of their pregnancies to all pregnant women during ANC, and (3) consumption by pregnant women of a daily dose of 1 g of elemental calcium (two tablets of 500 mg each) beginning at 4 months’ gestation (i.e., the timing of the first ANC visit as recommended by the MOHP and the median months’ gestation in the 2011 Nepal Demographic and Health Survey) for 5 months (150 days) or until delivery. Women were instructed to take two tablets (dose) once daily (frequency) in the morning (timing) for five months (duration). The daily dose of 1 g was based on the trials included in the 2010 Cochrane review available during study design [3]. Because of concerns that calcium might interfere with iron absorption [13], the women were instructed to take the calcium tablets after their morning meal (whereas iron was to be taken with the evening meal) and not to take it with iron. FCHVs were also expected to counsel pregnant women about calcium supplementation during their visits with them.

### District implementation

Calcium carbonate is registered with the Department of Drug Administration but is not on the national Essential List of Medicines. Jhpiego procured 26,500 bottles of calcium, each containing 100 tablets of calcium carbonate US Pharmacopeia (1250 mg equivalent to 500 mg/tablet of elemental calcium). Calcium was purchased first from Missionpharma India (US$ 0.01/tablet or Nepali Rupees 0.65/tablet) and then from Curex Pharmaceuticals Nepal (US$ 0.016/tablet or Nepali Rupees 1.35/tablet). Both shipments were delivered to the district health office, entered into the logistics management information system, and distributed to health facilities. Procured calcium was packaged in 100-tablet bottles, so three bottles was the standard amount distributed to women attending ANC at 4 months (total of 150 doses of 1 g elemental calcium each).

A district-level orientation was conducted for government district officials and health staff from nongovernmental organizations. All government health workers who provide ANC or supervise ANC staff (*n* = 268) and all FCHVs in the district (*n* = 810) were trained for 1 day by MOHP and MCHIP/Jhpiego staff. An educational poster about calcium supplementation during pregnancy was provided to all health facilities, and the birth preparedness package flipchart used by FCHVs was supplemented with an additional page of calcium messages.

After training, ANC providers began to educate pregnant women and distribute calcium, primarily during the first ANC visit. The intervention continued for a 14-month period from June 2012 to August 2013. The number of calcium bottles distributed to a pregnant woman was based on gestational age at her first ANC visit and the remaining period of her pregnancy (i.e., a pregnant woman coming at the 4th or 5th month of gestation was provided 300 tablets; those coming at 6 or 7 months were given 200 tablets; and those who came at 8 or 9 months received 100 tablets). The full supply of calcium was distributed at one visit in a small bag with an educational brochure. FCHVs met with pregnant women in their catchment area in the course of their normal duties, and were expected to encourage them to make ANC visits and also to educate them about calcium. FCHVs recorded basic information on calcium consumption during the postpartum visits they routinely conducted.

Monitoring of this intervention was integrated into the existing health management information system (HMIS). FCHV record forms were modified to capture information on calcium. A new calcium register was introduced to record distribution by ANC providers.

The district health office staff, with the support of two Jhpiego/MCHIP district-based staff, conducted periodic technical support visits to health facilities and FCHVs to monitor implementation of the program, reinforce key messages and address data collection issues. Updates were provided to the MOHP and the technical advisory group throughout the study to inform plans for national rollout.

### Study design

The study consisted of two components: prospective collection and secondary analysis of monitoring data captured by the MOHP through the district HMIS and a post-intervention household survey of women who had delivered in the last 6 months, or “recent mothers.”

### Sampling

Sample size calculations for the household survey of postpartum women were based on estimating ANC coverage in the general population of pregnant women and among women who received calcium, as well as compliance with the recommended calcium regimen among women who received calcium. Calculations used a one-sample proportion with high precision (5 %), including a design effect of 2 for the cluster design and an estimated nonresponse rate of 10 %. A total sample of 1230 postpartum women was required to achieve the desired degree of precision.

The household survey employed a three-stage cluster sampling approach to select women who had given birth in the last 6 months: the first stage selected VDCs randomly and the second stage using selected wards (nine wards per VDC; an average of 97 households per ward and an average of 5.3 persons per household) as the units of clustering. From the list of clusters, 62 clusters were chosen following the probability proportional to population method, with a target of 20 postpartum women per cluster to achieve a total of 1240. The interviewers visited every household of the sampled cluster to interview an eligible woman which entailed visiting an average of 10.4 households to find eligible woman.

### Data collection

Data from health facilities and FCHVs were collected monthly and analyzed to determine whether calcium was being distributed through ANC and consumed regularly by women. Data on pregnancy, delivery and calcium use collected on a monthly basis through FHCV registers and then summarized on health worker reporting forms were aggregated in monthly health facility reporting forms.

Post-intervention household interviews were conducted in August 2013 with women who had given birth in the last 6 months to measure overall calcium coverage, compliance with and acceptability of the recommended calcium regimen, along with ANC coverage. The questionnaire included the standardized Nepal DHS formulation of questions that were relevant to the calcium study. It was pretested and revised before data collection.

For the household survey, courtesy bias was minimized by hiring a local independent research agency, Population, Health and Development Group, instead of using project staff or government health officials, to conduct the interviews. The agency hired 24 field researchers, of whom 15 were women, who all received 1 week of training on maternal health, calcium intervention, study objectives, methodology and interview techniques. Training also included ethical considerations, mechanisms and techniques for confronting and overcoming their own biases, ways of administering specific questions, and probing strategies.

### Study measures

Information on compliance with the recommended calcium consumption regimen was captured in two ways. First, FCHVs conducted a tablet count at the end of pregnancy as part of routine monitoring. Second, we collected information on self-reported compliance in the household survey. The household survey also gathered information on correct dose, timing and frequency and duration of calcium consumption. Self-reporting is the international standard for measuring iron coverage and consumption through the DHS and the Multi-Indicator Cluster Survey (MICS) international household survey programs and thus it was the key method used in this study to measure calcium coverage and consumption during pregnancy.

Self-reported duration of calcium consumption also was measured. The number of days calcium was consumed was divided into three categories: a full course (150 days or 300 tablets), a partial course (90–149 days or 180–298 tablets), and short course (fewer than 90 days or less than 180 tablets). The number of days was determined using the responses from postpartum women who consumed calcium during their last pregnancy by asking, “How many days did you take calcium tablets during your last pregnancy?”

### Data analysis

A secondary analysis of the subset of the data in the health facility reporting forms, which is a compilation of the FCHV data, was entered into a database in Epi-Info and analyzed.

Descriptive analyses of the household survey data, including frequencies, means, medians and cross-tabulations using Pearson’s chi-square tests of significance, were conducted using SPSS v17. A p-value of 0.05 was used to determine statistical significance. Weights were applied to the sample to adjust for the urban/rural distribution of the population in Dailekh district [11], using the same procedure used in the Nepal DHS [8]. Multivariate logistic regression models, using robust (Huber-White) variance estimates that account for clustering, were fit to obtain the estimates in Table 5, using R statistical software v3.0.2 [14, 15]. Independent variables included in the multivariate analyses were the same as those that were included in the bivariate analyses, such as variables pertaining to personal characteristics of pregnant women that are known from the literature to affect health behavior in Nepal or other countries.

### Ethical approval

Ethical approval was obtained from Nepal Health Research Council and the Johns Hopkins University Bloomberg School of Public Health institutional review board. Informed verbal consent was obtained from the respondents to participate in the study.

## Results

### Sample characteristics

A total of 8641 pregnancies were expected for the 14-month program period (extrapolated from census data) [11]. The MOHP HMIS recorded 9246 pregnant women who came for ANC and received calcium. Women were mainly (98.8 %) from Dailekh district.

For the household survey, a total of 1240 postpartum women were interviewed, after screening 12,901 households. Study participants lived predominantly in rural areas, had low educational status (39 % with no education), were young (median age of 23), and were primarily of the Chhetri/Thakuri and Dalit castes (Table 1).

**Table 1:** Socio-demographic characteristics of women interviewed (*n* = 1240)

Characteristic	Number	Percentage
Residence		
Rural	1140	91.9 %
Urban	100	8.1
Age (in years)		
< 20	218	17.6
20–34	948	76.5
35–49	75	6.1
Median (mean)	23 (23.98)	
Education		
None	484	39.0
Primary	240	19.4
Some secondary	338	27.3
School leaving certificate and above	177	14.3
Caste/ethnicity^a^		
Chhetri/Thakuri	531	42.8
Bahun/Sanyasi	142	11.5
Janajati^b^	148	11.9
Dalit	420	33.9

^a^Caste/ethnicity stratification is a major determinant of people’s identity and social status in Nepalese society

^b^Janajati includes three Muslim groups and one Terai middle caste group

### Coverage of ANC and delivery services

Among the 1240 women interviewed in the household survey, 1173 (94.6 %) attended at least one ANC visit at a health facility during their last pregnancy. The median gestational age at the first ANC visit was 4 months (mean of 4.28 months). Most of the women (70.7 %) made four or more ANC visits during their most recent pregnancy. Urban women were more likely than rural women to attend ANC four or more times (90.0 % and 69.0 %, respectively). ANC was provided by a skilled provider (a doctor, nurse, to auxiliary nurse midwife) for 82.1 % of the women (Table 2).

**Table 2:** Coverage of ANC and delivery services among women interviewed

Characteristic	Number	Percentage
ANC among all women surveyed (*n* = 1240)		
Attended at least one ANC visit	1173	94.6
Attended at least one ANC visit with a skilled provider	1017	82.1
Attended four or more ANC visits	877	70.7
Urban (total *n* = 100)	90	90.0
Rural (total *n* = 1140)	785	69.0
Delivery care among women receiving calcium (*n* = 1173)		
Institutional delivery among women who received calcium	785	67.0

### Calcium coverage

All the women who attended ANC 1240 (100.0 %) were counseled on calcium use and received calcium. Calcium coverage (supplement distribution) therefore was 1173 (94.6 %) of all women surveyed. Among the women who received calcium (*n* = 1173), 965 (82.2 %) reported that they received 300 tablets, 161 (13.8 %) said they received 200 tablets, and 47 (4.0 %) reported receiving only 100 tablets (one bottle). A total of 73.0 % of the women who received calcium reported taking calcium beginning in the 4th month of pregnancy (Table 3). A total of 1157 women (93.3 %) received both calcium and iron.

**Table 3:** Amount of calcium received by gestational age at start of taking calcium (*n* = 1173)

Gestational age at start of taking calcium	Amount of calcium received					
	100 Tablets		200 Tablets		300 Tablets	
	Number	%	Number	%	Number	%
Month 3 (*n* = 88)	0	0	2	0.2	86	7.3
Month 4 (*n* = 856)	4	0.3	22	1.9	830	70.8
Month 5 (*n* = 60)	0	0	15	1.3	45	3.8
Month 6 (*n* = 118)	7	0.6	107	9.1	4	0.3
Month 7 (*n* = 31)	19	1.6	12	1	0	0
Month 8 (*n* = 17)	14	1.2	3	0.3	0	0
Month 9 (*n* = 3)	3	0.3	0	0	0	0

### Compliance with calcium intake instructions

Compliance with the recommended calcium supplementation instructions was examined by dose (two tablets), timing (after the morning meal), and frequency (daily). Among the women who received calcium, 99.2 % (*n* = 1164) reported taking the correct dose with the correct timing and frequency. Of the 1157 women who received both calcium and iron supplements, 99.8 % (*n* = 1155) reported taking them at separate times of the day, as instructed.

Compliance was further analyzed by the amount of calcium consumed. A full course is defined as 150 days (all 300 tablets), a partial course as 90–149 days (180–298 tablets), and a short course as less than 90 days (less than 180 tablets). Among the 1173 women who received calcium, 790 (67.3 %) reported that they completed a full course, 283 (24.1 %) reported taking a partial course, and100 (8.6 %) reported taking a short course. Full compliance for the intervention therefore was 67.3 %. For comparison, compliance data recorded by FCHVs as part of the regular program monitoring, were analyzed for the previous 6 months (the period covered by the survey). FCHV-reported data were consistent with the survey results: full course 67.6 %, partial course 22.7 %, and short course 8.4 %.

Ability to complete the full course was dependent on the gestational age at the time of calcium distribution (i.e., whether the pregnant woman received 300 tablets and had enough days before delivery to consume them all). Consumption was analyzed by the quantity of calcium received, and 91.4 % of women who received calcium consumed the entire course they were given. Among the 965 women who received the full course of calcium, 916 (73.8 % of all women surveyed) were in their 3rd or 4th month of pregnancy, so they could complete the full course of 150 days of calcium (Table 3). Among these, 768 women (83.8 %; *n* = 768/916) reported that they consumed the full course of 300 tablets in 150 days (Table 4). Therefore, 61.9 % (*n* = 768/1240) of women received the intervention as it was designed (i.e., effective coverage defined as daily consumption of 1 g of calcium for 150 days during pregnancy).

**Table 4:** Bivariate analysis of reported amount of calcium consumption by background characteristics of postpartum women household survey respondents (*n* = 1173)^a^

Background characteristics	Amount of calcium consumption (%)		
	Full course	Partial course	Short course
	(150+ days)	(90–149 days)	(<90 days)
Age			
< 20 (*n* = 208)	62.0	29.3	8.7
20–29 (*n* = 898)	69.0	22.7	8.2
30–45 (*n* = 67)	61.2	26.9	11.9
Residence			
Rural (*n* = 1075)	66.4	24.9	8.7
Urban (*n* = 98)	76.5	15.3	8.2
Education**			
No education (*n* = 443)	63.9	24.2	12.0
Primary (*n* = 227)	69.0	25.8	5.2
Some secondary (*n* = 328)	68.3	25.0	6.7
School leaving certificate and above (*n* = 175)	71.8	20.1	8.0
Gestational age received calcium***			
Month 3 (*n* = 88)	87.5	9.1	3.4
Month 4 (*n* = 856)	80.7	15.4	3.9
Month 5 (*n* = 60)	35.6	55.9	8.5
Month 6 (*n* = 118)	0.0	82.4	17.6
Month 7 (*n* = 31)	0.0	30.0	70.0
Month 8 (*n* = 17)	0.0	16.7	83.3
Month 9 (*n* = 3)	0.0	0.0	100.0
Caste/ethnicity			
Chhetri/Thakuri (*n* = 508)	68.6	23.8	7.7
Bahun/Sanyasi (*n* = 131)	67.9	24.4	7.6
Janajati (*n* = 134)	65.7	26.9	7.5
Dalit (*n* = 400)	66.0	23.5	10.5
Total (1173)	67.3	24.1	8.6

**p < .01, ***p < .001

^a^ *P*-values are based on Pearson’s chi square test of significance

There was some variation in calcium consumption by socio-demographic characteristics of the respondents and the gestational age at which they received and started taking calcium (Table 4). Cross-tabulations using a chi-square statistical test revealed significant differences in consumption by the mother’s age, education and gestational age at which calcium consumption began. A slightly higher proportion of women ages 20–29 and 30–45 than those less than 20 years of age completed a full course of calcium. A smaller proportion of women with no education than women with any education completed a full course of calcium. Caste did not appear to have any effect on calcium consumption.

Multivariate regression analyses were performed to further explore predictors of compliance with the recommended calcium intake regimen. These analyses were restricted to only those women who started taking calcium at 5 months or earlier in their pregnancy, so they could conceivably complete the recommended full course of calcium supplementation during pregnancy. Table 5 shows the results of the final multivariate logistic regression model, which includes all the covariates shown in the table, with each covariate adjusted for all the other covariates. The reference category for age is <20 years old. “Months pregnant” and “number of ANC visits” are modeled linearly as continuous variables. The reference category for “urban” residence is “rural” residence and the reference category for “education” is some, non-zero school attendance. The final model found that two variables are significant predictors of completing a full course (150 days) of calcium: gestational age at first ANC visit and total number of ANC visits attended. The estimated odds of completing a full course decreased with increasing gestational age, and increased as the total number of ANC visits increased. After adjusting for the other predictors in the model, the age of the mother was not found to be statistically significant, whether modeled as a linear effect, a linear effect with linear splines, or in three categories (<20, 20–29 and >30). Other multivariate models (data not shown) fit caste group as a predictor and included education modeled as a continuous linear predictor and in neither case did results change in the direction of any of the effect estimates or their statistical significance or non-significance. The size of the effect estimates also remained fairly consistent regardless of any of these changes with age, caste and education.

**Table 5:** Multivariate logistic regression analysis of predictors of a full course of calcium intake (150 Days) among women starting calcium by 5th month of pregnancy (*n* = 1036)^b^

Characteristics	Odds Ratio (Estimate)	95 % C I for ODDS ratio		Significance level
		lower	upper	*P*-value
Age 20–29	1.30	0.86	1.97	0.22
Age 30–45	1.12	0.66	1.92	0.67
No education	1.27	0.88	1.84	0.20
Month pregnant when made first ANC visit^a^	0.42	0.30	0.61	0.00*
Number of ANC visits^a^	1.74	1.36	2.21	0.00*
Urban	0.79	0.51	1.23	0.30

*p < .001

^a^continuous variables

^b^ *P*-values are based on Wald test results, and are based on the coefficient z-scores from the multivariate logistic regression, using robust (Huber-White) sandwich estimates of variance

### Discontinuation

About 10 % (*n* = 120) of the postpartum women said they stopped taking the calcium tablets before giving birth. The following reasons for discontinuation were reported: they became sick/ill (considered by the woman to be unrelated to calcium) they experienced side effects; they forgot to take the calcium; they found it difficult to take the tablets because they are too big; and they found it inconvenient to take the tablets every day.

### Knowledge about calcium and acceptability among postpartum women

Among all of the postpartum women who received calcium at ANC (*n* = 1173), 98.9 % (*n* = 1160) responded that pregnant women should take both iron and calcium tablets during pregnancy. Over 98 % of the women correctly answered questions on dose, timing, frequency and duration.

Several questions were included in the survey to assess the acceptability of calcium. Of the postpartum women who consumed any calcium (*n* = 1173), 983 (83.9 %) found the taste of the calcium acceptable; 1148 (97.9 %) would take calcium again in a future pregnancy; and 1144 (97.5 %) would recommend calcium to other pregnant women.

The study also examined whether ANC clients were routinely screened for PE/E. Among women who attended ANC and received calcium (n = 1173), 1150 (98.0 %) reported that they had their blood pressure measured at least once during ANC, and 1006 (85.7 %) had it taken at every ANC visit. Similarly, 1138 (97.0 %) said they had a urine test at least once, and 797 (67.9 %) said their urine was tested at every ANC visit.

### Feasibility of the intervention

There were no stock-outs of calcium reported, and all who attended ANC received calcium. By design, calcium was distributed at one visit to avoid issues with re-supply. Only 16 (1.4 %) of calcium consumers reported having difficulty storing the calcium. FCHVs were trained to provide education about calcium. A total of 953 of 1168 women (81.4 %) who received calcium (excluding five who were FCHVs) reported that FCHVs had visited them at least once during their last pregnancy. More than 99 % said they had received information on calcium use, iron use and ANC from the FCHV during these visits.

## Discussion

**Fig. 1:**
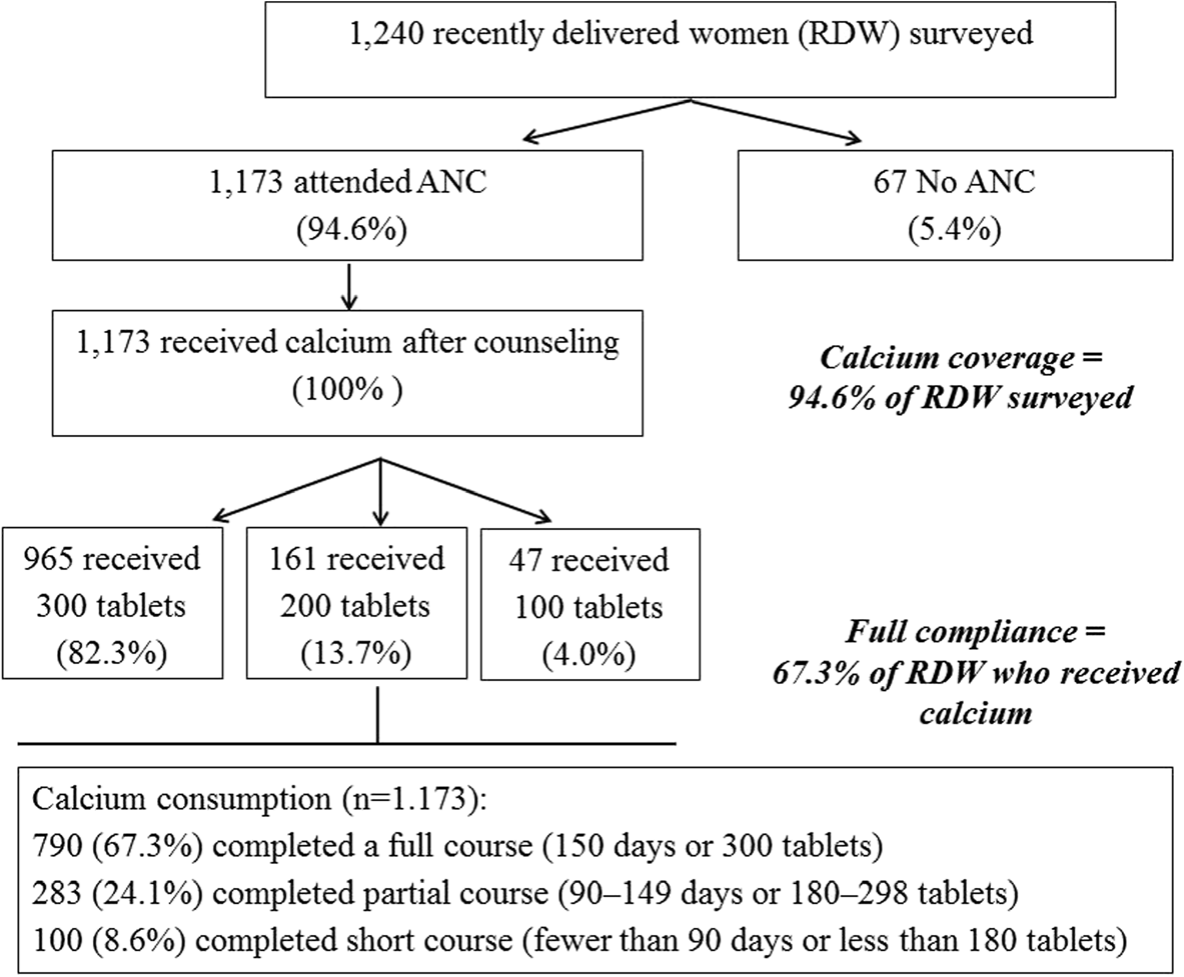
Flowchart of Calcium Distribution and Consumption

Overall, distribution of calcium through ANC services produced very high coverage of calcium supplementation among pregnant women—95 % of all postpartum women surveyed received calcium—along with high compliance—67 % of women who received calcium (61.9 % of all women surveyed) took the full course (Fig. 1). Moreover, the vast majority of all women who received calcium reported that they took it as instructed with respect to dosage, frequency and timing. This intervention was designed to provide 1 g of calcium daily for a total of 5 months, estimating that this amount, starting in the 4th month of pregnancy after the nausea of pregnancy had subsided, would take the client to term. The final regression analysis identified two significant predictors of completing a full course: gestational age at first ANC visit and number of ANC visits. These findings are logical in the sense that a greater number of ANC visits increases opportunities for additional education and reminders. In order to increase consumption of the full course of calcium to give pregnant women the maximum benefit from the intervention, calcium could be distributed at ANC visits in the first trimester, and consumption could be encouraged as early as feasible.

Surprisingly, ANC attendance in Dailekh was found to be higher than anticipated: 94.6 % of women made one ANC visit, and the proportion of women attending four or more ANC visits was much higher in Dailekh (70.7 %) than is reported nationally (50.1 %) [9]. The high coverage documented may be due to the fact that there were few private sector providers of maternal health service, so the government ANC platform proved to be the only option for ANC services. Likewise, the MOHP incentives for pregnant women to attend ANC provided by the Maternity Incentive Scheme might have contributed to the number of ANC visits early in pregnancy. With calcium in stock, ANC providers provided education and distributed calcium to 100 % of women attending ANC, demonstrating the feasibility of integrating calcium supplementation with ANC, without overburdening health care workers. The amount of calcium distributed correlated well with the gestational age of the pregnancy at the ANC visit during which the respondents received the calcium. Most of the women (73.8 %) received the full course of calcium by the 4th month of pregnancy, which allowed them enough time to consume the recommended course before birth. Encouragingly, nearly all women (99.8 %) who received both calcium and iron tablets reported taking them at separate times of day, so adverse effects of calcium supplementation on iron consumption and absorption were not of concern.

The majority of women (91.4 %) consumed all the calcium they received, and this high rate of compliance may have been due to the combined effects of proper counseling during ANC visits about the benefits of calcium in pregnancy; educational information received through multiple channels (poster, brochures, FCHVs); MOHP incentives for pregnant women to attend ANC early in pregnancy; and, potentially, the well-established FCHV-led iron distribution efforts in Nepal, which may have made taking a supplement familiar and acceptable to pregnant women.

Regarding the 10 % of the women surveyed who discontinued use of the calcium supplements, this level of discontinuation suggests a need for more strategic behavior change communication interventions at the community level, such as regular reminders from FCHVs or mobile text messages. Despite concerns about the size of the tablets, the chalky taste and the need to take multiple pills each day, calcium was found to be acceptable to pregnant women.

The overall design of the intervention was found to be feasible given that 100 % of clients making ANC visits were provided calcium during ANC; screening services were regularly provided at ANC visits; women reported no problem with storing calcium; and FCHVs and health workers played their roles well. The challenge moving forward may be procurement of the supplements at an affordable price.

There are several limitations to this study. Self-reported household survey data can be subject to recall bias; however, the recall period for questions pertaining to ANC experience was much shorter (up to 6 months) in this study than in international household surveys such as the DHS, which has a recall period of up to 5 years, and the MICS, with a recall period of up to 2 years. Thus, the quality of the self-reported household survey data in this study was expected to be high. Reported compliance was also verified with monitoring data collected by FCHVs. Data such as socioeconomic characteristics were not collected on women who did not attend ANC. The study was not designed to (1) collect serum calcium level (bio-marker) data to validate self-reported consumption; (2) collect qualitative data to explain the reasons for discontinuation or for late ANC attendance; or (3) measure the reduction in PE/E prevalence in the population.

When this study was designed, there was limited evidence on the minimum effective dose and duration of calcium supplementation. Recent recommendations suggest the daily dose may be reduced (e.g., 1.0–1.5 elemental calcium/day or 500–600 mg if high-dose supplementation isn’t feasible) and should be divided for ≤500 mg per dose [16–18]. This would prove significant in halving commodity costs, reducing storage needs and easing consumption. Omotayo et al. also suggest calcium and IFA pills be taken together to simplify patient instructions and improve adherence [16].

## Conclusions

This study found calcium supplementation during pregnancy, distributed through ANC, achieved the high levels of coverage and compliance. It was acceptable to pregnant women and feasible for the district public health system to distribute through existing public health facilities and health workers. These findings suggest the intervention can be expanded through ANC in Nepal. However, more efforts are needed to encourage pregnant women to attend ANC earlier to receive the full course of calcium supplementation.

## Abbreviations

ANC: Antenatal care
DHS: Demographic and Health Survey
FCHV: Female Community Health Volunteer
HMIS: Health Management Information System
MCHIP: Maternal and Child Health Integrated Program
MICS: Multi-Indicator Cluster Survey
MOHP: Minister of Health and Population
PE/E: Pre-eclampsia/eclampsia
VDC: Village Development Committee
